# Maternal and Perinatal Factors That Influence Susceptibility to Childhood Infections

**DOI:** 10.1097/INF.0000000000004865

**Published:** 2025-05-29

**Authors:** Isobel M. F. Todd, Wen-Qiang He, Natasha Nassar, David P. Burgner

**Affiliations:** From the * Murdoch Children’s Research Institute, Parkville, Victoria, Australia; † Department of Paediatrics, The University of Melbourne, Parkville, Victoria, Australia; ‡ Child Population and Translational Health Research, Children’s Hospital Westmead Clinical School, Faculty of Medicine and Health; § Leeder Centre for Health Policy, Economics and Data, Faculty of Medicine and Health; ¶ Charles Perkins Centre, The University of Sydney, Camperdown, New South Wales, Australia; ∥ Department of General Medicine, Infectious Diseases, Royal Children’s Hospital Melbourne, Parkville, Victoria, Australia; ** Department of Paediatrics, Monash University, Clayton, Victoria, Australia; †† Faculty of Health, Deakin University, Burwood, Victoria, Australia.

**Keywords:** infection, infectious disease, prenatal, pregnancy, birth, childhood

Understanding the drivers of the marked interindividual differences in susceptibility and severity of childhood infections is key to reducing its considerable burden, which occurs predominantly in the first 2 years of life. Numerous studies have investigated maternal and perinatal exposures and risk of infection in children. However, these findings can be difficult to interpret as childhood infections are a heterogeneous outcome with variation in rates across child’s age, regions of the world and clinical severity of disease. Additionally, outcome definitions may be pathogen-specific (eg, influenza), clinical syndromes (eg, pneumonia) or broad clinical categories of infection (eg, respiratory infections).

Effects of maternal and perinatal exposures are usually analyzed using observational study designs because it would be unethical or not feasible to undertake randomized controlled trials. Registry-based studies (analyzing routinely collected administrative data) are valuable tools in perinatal epidemiology as data collected in birth registries can be linked to other health registries (eg, hospital and prescription data). Prospective birth cohort studies are also commonly used in this context. Identifying causal mechanisms is challenging given the observational nature of these studies, as well as the interrelatedness of maternal and perinatal factors with each other and with individual and social determinants of health.

Here, we summarize the major epidemiological findings relating to associations between maternal and perinatal exposures and infections in children and highlight potential underlying mechanisms. We focus on maternal and perinatal exposures including (1) maternal health, including ongoing health conditions or lifestyle factors during pregnancy; (2) maternal medications during pregnancy; (3) maternal environmental pollutant exposure; (4) pregnancy- and birth-specific conditions or events and (5) infant growth parameters at birth and infant feeding (Fig. 1). We discuss their reported effects on childhood infections with a focus on overall and/or major clinical types of infections and findings from systematic reviews and population-based studies. Most studies are conducted in high-income countries. We did not limit by child age or infection severity; however, infection burden peaks in the first few years of life.

**FIGURE 1.**
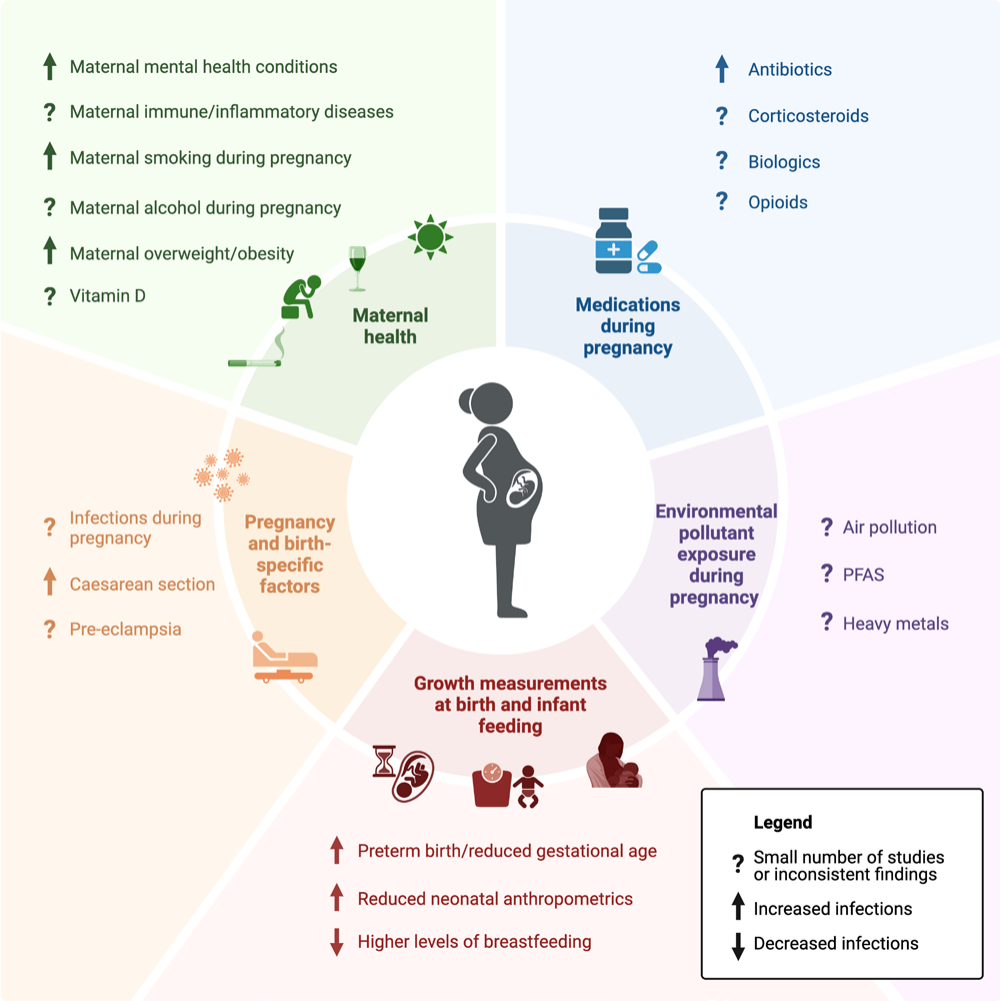
Summary of research on maternal and perinatal factors and their influence on childhood infections. Figure created in BioRender.

## MATERNAL HEALTH

Several population-based cohort and registry-based studies have explored and show a relatively consistent pattern of increased risk of infections in children of mothers with major mental health conditions. These include common (eg, depression, anxiety, stress and parental relationship distress) and rarer (eg, bipolar, schizophrenia and personality disorders) conditions.^1,2^ It is unclear the extent to which these associations represent an *in utero* biological effect (eg, dysregulation of the maternal hypothalamic pituitary adrenal axis) and/or postnatal behavioral/social mechanisms.

In largely single population-based studies, various maternal immune and inflammatory diseases (eg, maternal celiac disease^3^ and inflammatory bowel disease^4^) have been associated with childhood infections. The mechanisms are poorly understood and may include immunological differences in offspring arising from the underlying condition or immunomodulatory therapy. Further studies to replicate findings and separate the effects of the underlying condition and/or medication are required.

In terms of maternal lifestyle factors, several studies have shown an association between maternal tobacco smoking during pregnancy and higher infection risk in offspring.^5,6^ Potential mechanisms include an increased risk of preterm birth and low birthweight, resulting in reduced lung function at birth and suboptimal immune development.^6^ Maternal smoking during pregnancy is also correlated with paternal and household tobacco smoke exposure, amplifying infection risk postnatally. The limited studies on prenatal alcohol exposure and infections generally indicate increased risk, although this relationship is less explored.^7^ Maternal overweight and obesity have been shown to confer an increased infection risk.^8,9^ These effects may be partially mediated through factors, which occur at higher rates in pregnancies in those with overweight/obesity, including preeclampsia, cesarean section and preterm birth, or may relate to effects of obesity on immune responses. Smoking, alcohol use and overweight/obesity all have complex determinants and a strong socioeconomic gradient. Many of these drivers are difficult to measure and some residual confounding is likely. Notwithstanding, interventions should aim to minimize these exposures, even if their relative contributions are less clear.

The relationship between vitamin D levels during pregnancy and/or at birth and childhood infections has been studied extensively, particularly for lower respiratory tract infections. Several systematic reviews have summarized findings from observational studies on maternal and/or infant cord blood vitamin D concentration and randomized trials of vitamin D supplementation during pregnancy. These reviews report modest protective to null effects of higher vitamin D levels and supplementation on offspring infections.^10–12^

## MEDICATIONS DURING PREGNANCY

Antibiotics are the most prescribed medication during pregnancy and their use has received attention regarding possible impacts on childhood infection risk. Total-population studies have found increased risk of severe childhood infections (requiring hospitalization or specialist outpatient care) following antibiotic exposure during pregnancy,^13,14^ in addition to increased rates of antimicrobial prescriptions and infection-related mortality in the first year of life.^14^ Limited evidence on the effects of intrapartum antibiotic exposure shows a potential small increased infection risk.^15^ A common proposed mechanism is dysbiosis of the maternal and consequently the inherited infant microbiome following antibiotic use. It is also suggested that these associations may be explained by confounding by indication—where the indication for a certain exposure is responsible for the observed effect, in this case maternal infections during pregnancy, rather than the exposure itself—or by other residual confounding (as suggested by sibling analyses^14^).

Immunomodulatory medications, which can cross the placenta, may be prescribed for either maternal conditions or to improve neonatal outcomes in high-risk pregnancies, and may affect offspring infection risk. Given the increased risk of severe infection with corticosteroid exposure in adults and children, there is interest in whether antenatal corticosteroids influence child infection risk. Corticosteroids are recommended to promote fetal lung maturation in pregnancies at-risk of preterm birth. In a total-population study in Taiwan of 2 million children (2.3% exposed), a 24% increased risk of serious infections in the first year of life was observed in those exposed to corticosteroids.^16^ The effect sizes were greater in infants born at term (compared with preterm), possibly reflecting the difference in baseline infection risk with gestational age of the comparison groups.

Biologics are increasingly used in pregnancy. Most data on the putative effects on offspring are from older biologics, such as tumor necrosis factor inhibitors. In large registry studies comparing exposure to anti-tumor necrosis factor biologics in pregnancy with healthy unexposed pregnancies, there is evidence of increased risk of offspring hospitalized infections in the first year of life. Where the comparator group includes those with the underlying inflammatory condition (eg, inflammatory bowel disease), the findings are less consistent, possibly reflecting the underlying inflammatory condition in the mother, and/or exposure to nonbiologic immunomodulatory medication (eg, corticosteroids).^17^

There is increasing interest in opioid use during pregnancy and offspring health outcomes. In a Scandinavian multicountry study, women who continued opioid maintenance therapy during pregnancy were compared with women who discontinued opioid use and women with opioid analgesic use for more than 1 month of pregnancy were compared with short-term opioid analgesic use. Neither of the 2 comparisons showed an increased risk of antibiotic prescriptions or hospitalized infections in offspring.^18^ In contrast, an Australian registry study showed increased risk of hospital presentations/admissions for infections in children following analgesic opioid use during pregnancy but not following opioid maintenance therapy.^19^

## MATERNAL ENVIRONMENTAL POLLUTANT EXPOSURE

There is increasing recognition of potential adverse health effects arising from maternal exposure to environmental pollutants, particularly air pollution, synthetic organic chemicals and heavy metals. Exposure to these chemicals during pregnancy may exert toxic effects on organogenesis and immune development.

Air pollution exposure studies have focused on risk of respiratory infections. Suggested mechanisms include direct effects of pollutants on organ development, inflammation-associated pathways and indirectly through increased risk of adverse events such as preterm birth. Higher levels of postnatal air pollution exposure are linked with increased lower respiratory tract infection risk,^20^ but the evidence around prenatal exposure remains inconclusive.^21^ Fewer studies isolate the specific effect of prenatal air pollution exposure, which is difficult given its correlation with postnatal exposure.

A systematic review and meta-analysis of 12 studies on exposure to per- and polyfluoroalkyl substances (PFAS) (most measured during pregnancy) and immune outcomes assessed associations between 10 individual PFAS chemical compounds and 12 infection outcomes. Most associations were minimal with confidence intervals inclusive of the null. Some positive associations, albeit all from single studies, were observed with certain PFAS and any infection, respiratory infections, pseudocroup and otitis media.^22^ However, further studies are required.

There is some evidence suggesting that increased exposures to toxic metals (eg, lead, mercury, arsenic and cadmium) are associated with adverse effects on immune biomarkers, weaker responses to vaccination and increased risk of allergies and infections, although findings are inconsistent and complicated by varying exposure windows and measurement methods.^23^ There are few studies that specifically consider toxic metal exposure prenatally and its effects on child infection outcomes. Three studies concerning prenatal arsenic exposure showed potential increased risks of respiratory infections and diarrhea.^23^ Further studies addressing prenatal exposure and offspring infections are required.

## PREGNANCY AND BIRTH-SPECIFIC FACTORS

It is suggested that acute maternal infection during pregnancy may affect offspring immune responses, either directly or via antibiotic-mediated effects as discussed above. However, there are few epidemiological studies on long-term effects on childhood infection. While findings of increased risk of childhood infection were consistent, these studies investigated different maternal infection types including maternal urinary tract infection during pregnancy,^24^ maternal hospitalizations for infections during pregnancy^25^ and maternal infection during pregnancy based on medical records.^26^

There has been considerable interest in the mode of birth and its association with offspring infection. A recent systematic review including data from over 10 million births found that cesarean section birth was associated with a 10%–20% increased risk of hospitalization for childhood infections and with other, less severe infection outcomes.^27^ A commonly proposed mechanism is differences in the newborn microbiome by mode of birth. However, there is potential for confounding by indication in such studies from maternal health conditions and lifestyle factors associated with cesarean section.

A single study on preeclampsia found that although rates of infection-related hospitalization were higher in children born to pregnancies complicated by severe preeclampsia, there was no evidence of an independent effect of severe preeclampsia after adjusting for cesarean section, intrauterine growth restriction and gestational age.^28^

## GROWTH MEASUREMENTS AT BIRTH AND INFANT FEEDING

Preterm birth/early gestational age and reduced infant anthropometry (birth weight and length) are consistently associated with a higher risk of infections in childhood. Several registry-based studies have demonstrated increased risk of hospitalized infections for children born preterm and early term or with lower birth weight and length.^29,30^ These associations are highest at the extremes but are present across the entire range of suboptimal neonatal anthropometrics and gestational age and persist into late adolescence.^30^ Interaction analyses highlight that those born preterm and small for gestational age are a particularly high-risk cohort of children.^29^

Breastfeeding has a well-documented protective role against childhood infectious diseases across high-, middle- and low-income countries. In the most comprehensive review to date,^31^ meta-analyses of greater versus less breastfeeding (eg, exclusive vs. nonexclusive; predominant vs. partial; partial vs. none and any breastfeeding vs. no breastfeeding) showed decreased diarrhea incidence and hospitalization in children under 5 years, and reduced lower respiratory tract infection incidence/prevalence and hospitalization, acute otitis media and infectious disease mortality in children under 2 years. Breast milk contains numerous beneficial components (eg, antibodies, lactoferrin, oligosaccharides, cytokines and growth factors), which may impact a child immune function both directly and indirectly (eg, by effects on the microbiome).

## DATA CONSIDERATIONS AND LIMITATIONS

Most of the abovementioned maternal and perinatal exposures are difficult and/or unethical to investigate in human experimental studies. As such, findings in this review are largely from observational epidemiological studies, where potential unmeasured confounding is a ubiquitous issue. Many were total-population studies using routine administrative health registry data, which are advantageous as they have large sample sizes leading to high precision; minimize selection bias from participation and loss-to-follow-up; and the findings are more generalizable to target populations. However, confounding bias is not eliminated by these designs. In addition, the field may be prone to publication bias, where positive/negative associations are more likely to be published than null findings. In this scenario, exposure-outcome associations reported in meta-analyses would be greater in magnitude or appear more conclusive than is the reality. It is important to be cognizant of these biases when interpreting these types of epidemiological studies.

## CONTEXT CONSIDERATIONS AND FUTURE DIRECTIONS

We have highlighted the diverse maternal and perinatal factors that may influence offspring infections. These factors have complex genetic, environmental and social determinants, and many are interrelated. The concept of risk factor clustering—where the observed prevalence of a combination of risk factors is greater than expected from their individual prevalence—is pertinent for maternal and perinatal exposures, which usually occur together. Some relationships are directly causal (eg, maternal smoking during pregnancy and lower birthweight), while others are only correlations, for example, both exposure to environmental pollutants and maternal overweight/obesity may be higher in individuals with greater social disadvantage. Most studies summarized here adopt a causal framework and therefore focus on single exposure-outcome associations, adjusting for relevant confounders. This obfuscates the potential effects of risk factor clustering, as often occurs in reality. Risk factor clustering is important to consider for subgroups of children who may be at highest risk of severe infections. Risk-stratification may guide targeted preventative interventions and management of specific at-risk groups to reduce postnatal infection burden.

To bridge the gap between primary epidemiological findings and public health interventions, further research addressing mediation pathways and risk prediction are warranted. In addition, mechanistic studies of biological pathways (eg, effects of exposures on the microbiome, epigenetic changes, impacts of dysregulation in hormonal pathways or inflammation) would inform possible interventions. Concurrently, primordial prevention efforts should target known, modifiable risk factors at an individual and community level. Most studies are from high-income countries, and further research in low- and middle-income countries, where infection burden is greatest, is essential.
